# IFNγ and TNFα drive an inflammatory secretion profile in cancer‐associated fibroblasts from human non‐small cell lung cancer

**DOI:** 10.1002/1873-3468.15083

**Published:** 2025-01-01

**Authors:** Lilian Koppensteiner, Layla Mathieson, Liam Neilson, Richard A. O'Connor, Ahsan R. Akram

**Affiliations:** ^1^ Centre for Inflammation Research, Institute for Regeneration and Repair University of Edinburgh UK; ^2^ Cancer Research UK Scotland Centre, Institute of Genetics and Cancer The University of Edinburgh UK

**Keywords:** cancer‐associated fibroblast, interferon‐licenced fibroblast, non‐small cell lung cancer, secretome, tumour microenvironment

## Abstract

Cancer‐associated fibroblasts (CAFs) are the dominant nonmalignant component of the tumour microenvironment (TME). CAFs demonstrate a high level of inter‐ and intra‐tumour heterogeneity in solid tumours, though the drivers of CAF subpopulations are not fully understood. Here, we demonstrate that non‐small cell lung cancer (NSCLC) patient‐derived CAFs upregulate the secretion of inflammatory cytokines (IL6, LIF, IL33, GM‐CSF, IL1ra) and chemokines (CCL2, CCL3, CCL4, CCL20, CXCL8, CXCL9, CXCL10, CXCL11) in response to *in vitro* co‐culture with anti‐CD3/anti‐CD28‐stimulated peripheral blood mononuclear cells (PBMCs) via IFNγ and TNFα. Furthermore, T‐cell‐derived IFNγ inhibits CXCL12 secretion by CAFs *in vitro*. Our results highlight the ability of T‐cell effector cytokines to modulate the CAF secretome in NSCLC.

## Graphical abstract

As the most abundant stromal cell type of the tumour microenvironment of non‐small cell lung cancer (NSCLC), cancer‐associated fibroblasts (CAFs) play a crucial role in modulating the immune landscape via multiple mechanisms. This study demonstrates that cytokines from effector T cells can induce and modulate the secretion of cytokines and chemokines by CAFs *in vitro*, highlighting the immunomodulatory capacity of CAFs and their potential to shape the immune response in NSCLC.

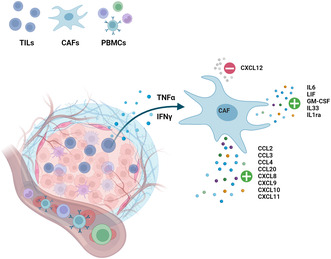

## Abbreviations

**ANOVA**, analysis of variance

**CAF**, cancer‐associated fibroblast

**CCL**, chemokine (C‐C motif) ligand

**CCR**, C‐C chemokine receptor

**ECM**, extracellular matrix

**ELISA**, enzyme‐linked immunosorbent assay

**ELLA**, enzyme‐linked lectin assay

**FAP**, fibroblast activation protein

**GM‐CSF**, granulocyte‐macrophage colony‐stimulating factor

**IFNγ**, interferon gamma

**IL**, interleukin

**LUAD**, lung adenocarcinoma

**LUSC**, lung squamous cell carcinoma

**NSCLC**, non‐small cell lung cancer

**PBMC**, peripheral blood mononuclear cell

**PDGFRβ**, platelet‐derived growth factor receptor beta

**PDPN**, podoplanin

**TCGA**, The Cancer Genome Atlas

**TCM**, T‐cell‐conditioned media

**TME**, tumour microenvironment

**αSMA**, alpha smooth muscle actin

Cancer‐associated fibroblasts (CAFs) make up the majority of the nonimmune tumour microenvironment (TME), profoundly influence the movement and behaviour of immune cells in the stromal niche and specific CAF phenotypes demonstrate diverse functions [1, 2]. Öhlund *et al*. [3] report two transcriptionally distinct subpopulations of CAFs in pancreatic cancer; inflammatory CAFs (iCAFs), which are a significant source of interleukin 6 (IL6), and myofibroblastic CAFs (myCAFs), with high expression of alpha smooth muscle actin (αSMA), a key role in extracellular matrix (ECM) remodelling and transforming growth factor beta (TGFβ) signalling. We have characterised CAF heterogeneity in non‐small cell lung cancer based on expression of αSMA as well as other stromal surface markers implicated in survival (e.g. platelet‐derived growth factor receptor beta (PDGFRβ), fibroblast activation protein (FAP), fibroblast specific protein 1 (FSP1)) and identified five CAF subsets (CAFS1–CAFS5), and similar phenotypes of CAFs have been identified and share characteristics across different solid tumours [4]. CAFs can respond to local autocrine and paracrine signalling from the microenvironment, which could influence CAF functions within a tumour. Interestingly, *in vitro* culture conditions can affect whether CAFs adapt an iCAF or myCAF profile, suggesting that CAF phenotypes can be transient and may represent a dynamic response to direct or indirect cell signalling interactions in the local environment [3]. Mechanistically, drivers of the development of specific CAF subsets and their secretory profile are not fully understood [5]. We have previously shown that activated T cells induce major histocompatibility complex (MHC) II expression and upregulate CD73 expression as well as IL6 and IL27 secretion in NSCLC CAFs *in vitro* via IFNγ and TNFα, reminiscent of the newly identified interferon‐licenced fibroblast [6, 7]. Here, we aimed to test whether NSCLC CAFs develop an interferon‐licenced fibroblast secretory profile in response to sensing activated T cells in their environment, which could have implications in immune cell recruitment and function in the TME of NSCLC.

## Materials and methods

### Ethics statement

All experiments using human samples were undertaken with the understanding and written consent of each subject. The study methodologies were approved by the regional ethics committee (REC). Healthy volunteer blood was obtained following informed consent, and the study was approved by Lothian Regional Ethics Committee (REC) (REC No: 20‐HV‐069) prior to enrolment in the studies. Peripheral blood and tumour tissue samples were obtained following written informed consent and approval by NHS Lothian REC and facilitated by NHS Lothian SAHSC Bioresource (REC No: 15/ES/0094). Samples were collected from September 2020 to April 2024 at the Centre for Inflammation Research and the Royal Infirmary Edinburgh. The study methodologies conformed to the standards set by the Declaration of Helsinki.

### Generation of CAF lines

Tissue samples from consented early NSCLC patients. Tissue samples were mechanically minced and chemically digested with 1 mg·mL^−1^ Collagenase IV (Merck) and 1 mg·mL^−1^ DNAse 1 (Merck, Darmstadt, Germany) in media for 1 h at 37 °C and passed through 70 μm filters. Red blood cells (RBC) were lysed with RBC lysis buffer (BioLegend, San Diego, CA, USA). The resulting single cell suspensions from tissue digests were plated in flasks in prewarmed Dulbecco's modified Eagle medium (DMEM) with 100 μL penicillin/streptomycin, 2 mm l‐glutamine and 10% FCS (all Gibco, Billings, MT, USA) and incubated at 37 °C. Nonadhering cells were washed away and DMEM with 100 μL penicillin/streptomycin, 2 mm l‐glutamine, 10% fetal calf serum (FCS) and 1× Insulin Transferrin Selenium (ITS) was added to the flasks. At passage, cells were washed in phosphate‐buffered saline (PBS) and treated with 0.5% Trypsin EDTA for 3 min at 37 °C to lift cells. At the third passage, CAF lines were cryopreserved. All CAF lines were used at passage 3–6.

### Peripheral blood mononuclear cells extraction and T‐cell isolation

Peripheral blood mononuclear cells (PBMCs) from healthy donors or NSCLC patients were isolated from whole blood using lymphoprep (Stemcell Technologies, Vancouver, Canada) according to manufacturer's instructions. T cells were purified by immunomagnetic‐negative selection using the EasySep Human T cell Isolation kit (Stemcell Technologies), in accordance with manufacturer's instructions.

### Generation of T‐cell conditioned media

T‐cell conditioned media (TCM) was generated as previously described by O'Connor *et al*. [7] First, tumour‐infiltrating lymphocytes were grown out from tumour sample sections and cultured in media containing IL2. Subsequently, T cells were plated in 12‐well plates at 5 × 10^5^ mL^−1^ and stimulated with anti‐CD3/anti‐CD28 (both 1 μg·mL^−1^ Bio‐Xcell/BioLegend) for 48 h. After 48 h, cells were collected and centrifuged at 350 ***g*** for 5 min and supernatants were harvested and filtered through 0.22 μm syringe filters and stored at −20 °C. T‐cell conditioned media were added to CAF cultures at a 1 : 1 ratio.

### CAF culture/co‐culture conditions

Cancer‐associated fibroblasts were seeded in 12‐well plates (Corning, NJ, USA) at 4 × 10^4^ cells/well and incubated at 37 °C. After 24 h of incubation, media was replaced with or without addition of 10 ng·mL^−1^ rIFNγ, 25 ng·mL^−1^ rTNFα or a combination of rIFNγ and rTNFα for a total culture volume of 1 mL. The concentration used to assess the effect of IFNγ is based on concentrations observed during CAF‐T cell co‐culture *in vitro* [7, 8] and rTNFα is used at concentrations with physiological relevance to cancer cells and fibroblasts [9, 10, 11]. Supernatants were collected after 48 h. For co‐culture experiments, CAFs were plated in 48 well plates at 2 × 10^4^ cells/well and allowed to adhere for 4 h prior to adding PBMCs or purified T cells at 5 × 10^5^/well in 1 mL for a total culture volume of 1 mL. PBMCs or purified T cells were stimulated with soluble anti‐CD3/anti‐CD28 (1 μg·mL^−1^; Bio‐Xcell/BioLegend), or anti‐CD3/anti‐CD28 coated beads (Dynabeads, Thermo Fisher Scientific, Waltham, MA, USA) at a 1 : 1 bead : cell ratio. Supernatants were collected after 96 h. All supernatants were centrifuged at 350 ***g*** for 10 min and stored at −20 °C.

### Secretome analysis

ELISAs were performed according to manufacturers' instructions for IL6 (BioLegend), LIF (R&D Systems, Minneapolis, MN, USA) and CXCL12 (R&D Systems) in technical duplicates. GM‐CSF, IL1ra, CCL2 and CXCL10 levels were analysed using the ELLA platform (Protein Simple). Data was calculated by the ella software (Bio‐Techne, Minneapolis, MN, USA) and corrected for dilution factors (internal triplicates). A set of seven analytes (IL33, CCL3, CCL4, CCL20, CXCL8, CXCL9, CXCL11) were quantified according to manufacturers' instructions, using a custom Legendplex‐assay (LEGENDplex™ BioLegend). Data collection was performed according to manufacturer's instructions using the Attune NxT Autosampler. Analysis was performed using LEGENDplex™ data analysis software.

### Flow cytometry

Cells were washed in PBS followed by staining of dead cells with Zombie Live Dead UV (BioLegend). Cells were treated with TruStain FcX (BioLegend) for 5 min prior to surface staining with the indicated monoclonal antibodies (Table 1) for 20 min at 4 °C. Subsequently, cells were washed in PBS and fixed in 2% PFA (BioLegend). All flow cytometry data were collected on a six laser LSR Fortessa (BD, Franklin Lakes, NJ, USA). Single stain controls were used to set the compensation prior to collection of FACS data. Data were analysed using flowjo software (BD).

**Table 1:** Flow cytometry staining panel.

Antibody	Fluorophore	Source
Epcam	BV605	BioLegend
CD45	BV605	BioLegend
CD31	BV605	BioLegend
FAP	APC	R&D Systems

### Single cell RNA sequencing analysis

Open‐source data from Grout *et al*. [12] were analysed using R. The fibroblast data set was filtered for fibroblasts that could be defined as myCAFs or iCAFs based on the expression of the following markers at above median counts across the whole fibroblast dataset: myCAFs: FAP, ACTA2, HOPX, MYH11 and POSTN, and iCAFs: IL6, CXCL12 and PDGFA. Differential expression analysis was then performed in R using the DESeq2 package to extract the normalised counts [13]. Normalised counts of the markers of interest were then plotted and compared between CAF subsets.

### Analysis of TCGA data

Data for primary lung cancer (adenocarcinoma (TCGA‐LUAD) and squamous cell carcinoma (TCGA‐LUSC)) were downloaded from https://tcga‐data.nci.nih.gov. The median function in R was used to determine a cut‐off across the whole dataset for each of the markers FAP, PDPN and αSMA. Using these cut‐offs generated patients could be defined as low or high for each marker. Patients were classified as being predominant for a specific CAF subtype by the following definitions: CAF‐S1: FAP^high^, PDPN^high^ and αSMA^high^; CAF‐S4: FAP^low^, PDPN^low^ and αSMA^high^; CAF‐S5: FAP^high^, PDPN^high^ and αSMA^low^; and patients were defined as ‘other’ if none of these criteria were met. The counts of each gene of interest were then extracted for all the patients and compared across the subtypes.

### Statistical analysis

One‐way analysis of variance (ANOVAs) with Tukey's multiple comparisons were used to compare multiple experimental groups (ns, not significant; *P ≤ 0.05, **P ≤ 0.01, ***P ≤ 0.001).

## Results

### PBMC – CAF crosstalk promotes cytokine and chemokine release in the TME of NSCLC

**Fig. 1:**
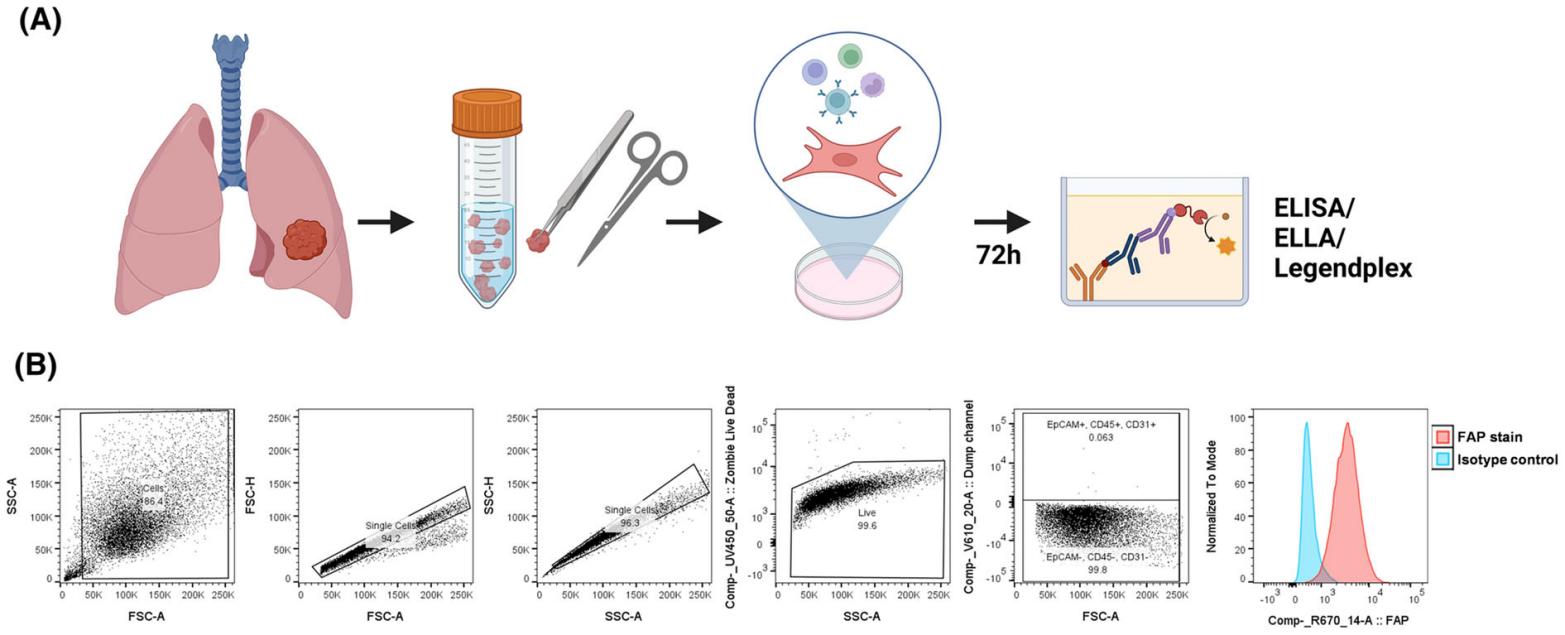
CAFs isolated from NSCLC tumour tissue are FAP^+^ during *in vitro* culture. (A) Preparation of NSCLC tumour samples for *in vitro* expansion of CAFs and downstream secretome analysis. (B) Representative flow cytometry staining of CAFs *in vitro*. Image created with BioRender.

To assess the CAF secretome response to inflammatory stimuli, CAFs were isolated from tumour tissue digests of early NSCLC patient resections and cultured *in vitro* (Fig. 1A). Flow cytometry analysis confirmed that *in vitro* cultured CAFs were negative for lineage markers for epithelial cells (Epcam), leucocytes (CD45) and endothelial cells (CD31) and positive for the CAF marker fibroblast activation protein (FAP) (Fig. 1B).

**Fig. 2:**
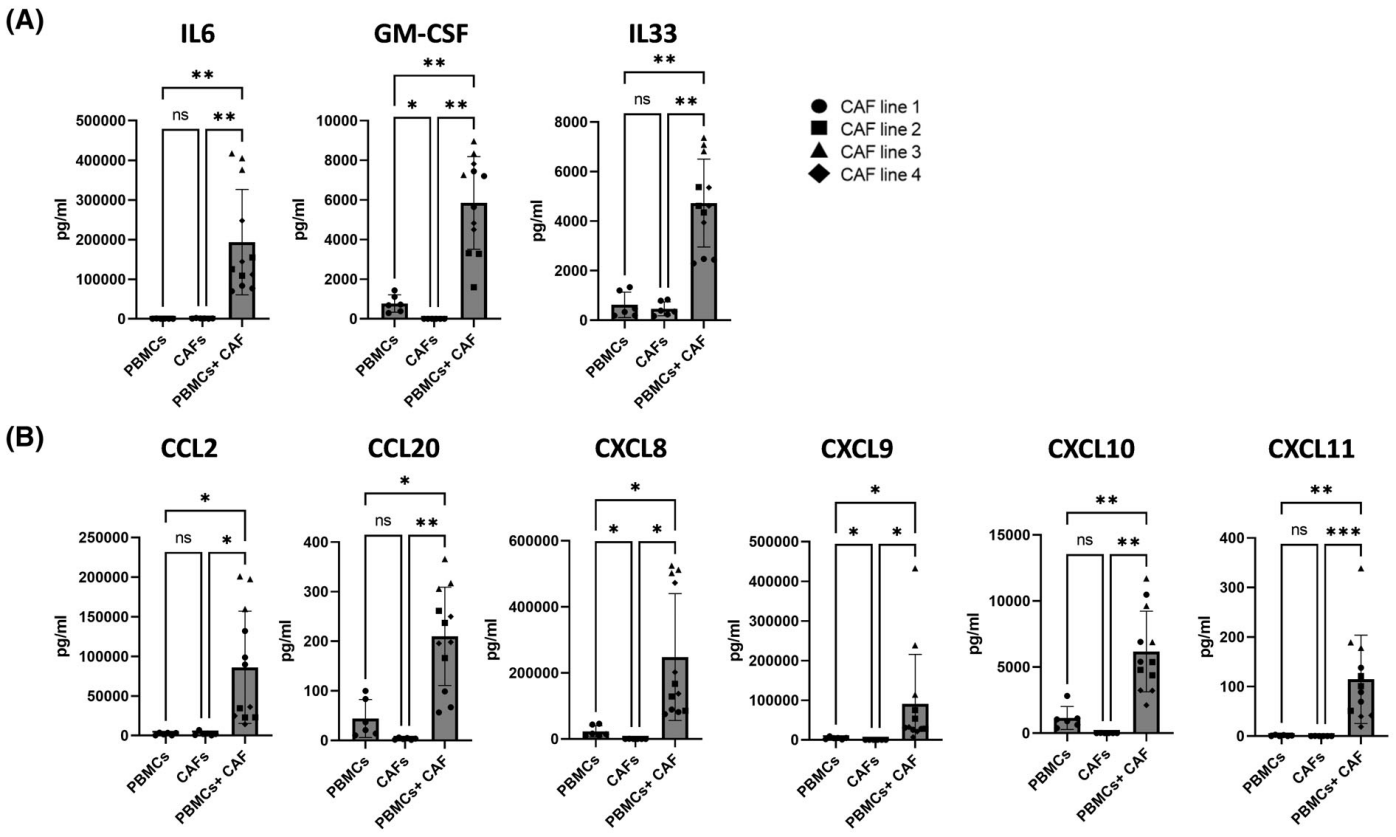
CAF‐T cell interactions increase secretion of cytokines and chemokines. PBMCs from three healthy donors were stimulated with anti‐CD3/anti‐CD28 (PBMCs). Four independently generated NSCLC CAF lines were cultured alone (CAFs) or in the presence of anti‐CD3/anti‐CD28 stimulated PBMCs (PBMCs + CAFs). (A) Production of IL6, IL33 and GM‐CSF production and (B) CCL2, CCL20, CXCL8, CXCL9, CXCL10 and CXCL11. One‐way ANOVA was used for all statistical analysis with Tukey's multiple comparisons test (ns, not significant; *P ≤ 0.05, **P ≤ 0.01, ***P ≤ 0.001). Error bars show standard deviation of the mean. *n* = 4 CAF lines.

To determine the effect of T cells on the CAF secretome, CAFs were co‐cultured with healthy donor PBMCs treated with anti‐CD3 and anti‐CD28 to activate T cells. Co‐culture of CAFs and PBMCs resulted in a strong IL6 response, consistent with previous reports [7]. Similarly, IL33, which is enriched in the iCAF cluster of a murine breast cancer model [6], is also significantly increased during PBMC – CAF co‐culture (Fig. 2A). We additionally aimed to assess GM‐CSF as a known secreted factor involved in CAF‐mediated immunomodulation [2, 14]. While PBMCs produce minimal GM‐CSF, and CAFs alone produce none, we observed a ~ 10‐fold increase during co‐culture (Fig. 2A). We also assessed the production of chemokines during co‐culture, as high expression of genes related to chemokine production is a main feature of iCAFs and ilCAFs [3, 5, 6, 15]. While untreated CAFs do not produce any chemokines, the presence of stimulated PBMCs caused a significant increase in CCL2, CCL20, CXCL8, CXCL9, CCXCL10 and CXCL11 (Fig. 2B). These data were reproduced with PBMCs from early NSCLC patients, demonstrating that PBMCs from NSCLC patients drive similar chemokine and cytokine release in co‐culture with NSCLC‐derived CAFs (Fig. S1).

In addition, co‐culture of CAFs with anti‐CD3/anti‐CD28 stimulated purified T cells isolated from healthy donor PBMCs mirrored this data, suggesting that T cells are responsible for these changes (Fig. S2). Taken together, the above data illustrate that crosstalk of NSCLC CAFs and T cells results in a significant increase in the production of cytokines and chemokines related to an iCAF phenotype.

### IFNγ and TNFα drive cytokine and chemokine release by NSCLC CAFs

**Fig. 3:**
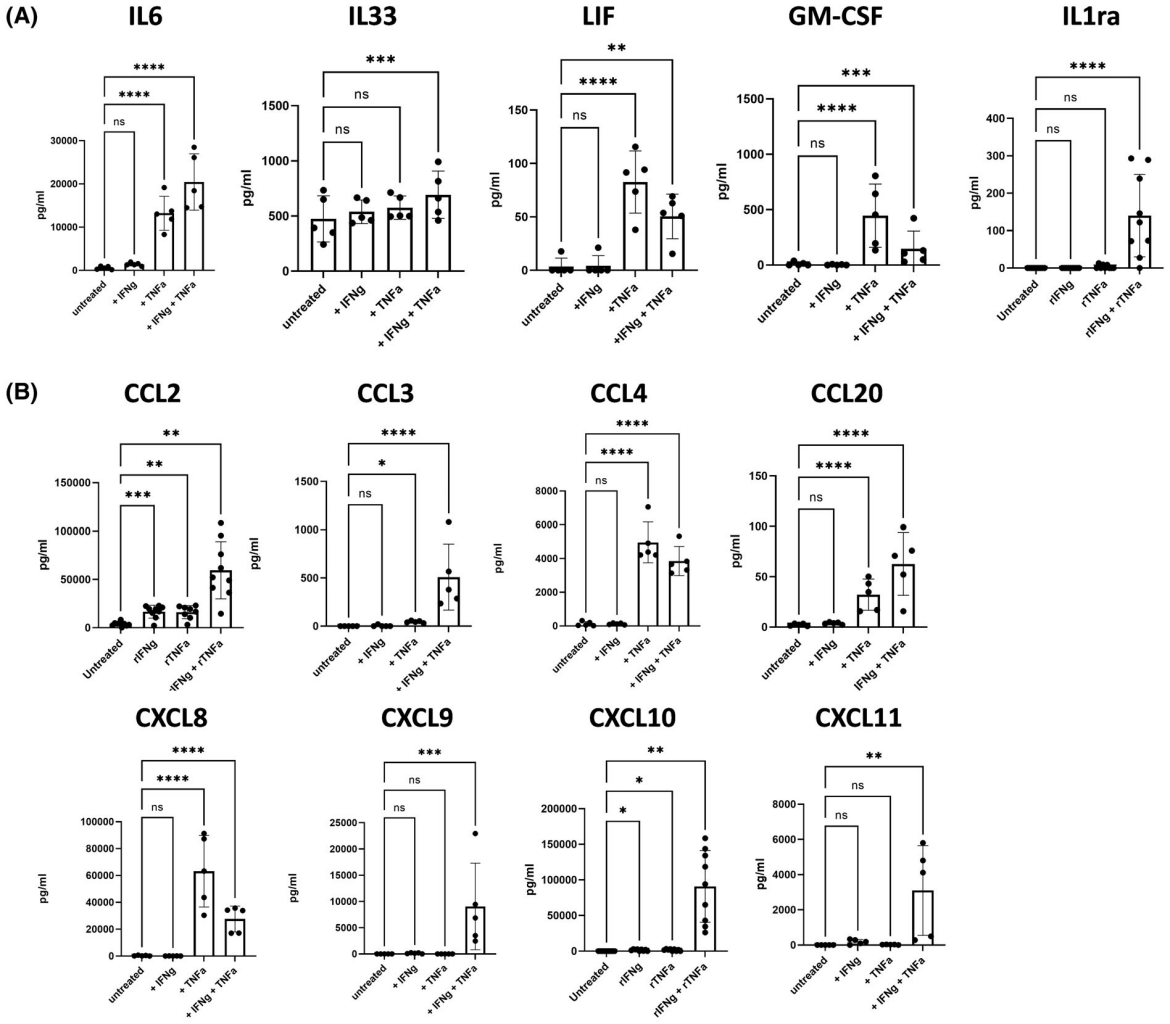
IFNγ and TNFα induce secretion of cytokines and chemokines by CAFs. NSCLC CAF lines were treated with recombinant rIFNγ (10 ng·mL^−1^), TNFα (25 ng·mL^−1^) or a combination of rIFNγ and TNFα. (A) Production of IL6 (*n* = 5), IL33 (*n* = 5), LIF (*n* = 5), GM‐CSF (*n* = 5) and IL1ra (*n* = 9) and (B) CCL2 (*n* = 9), CCL3 (*n* = 5), CCL4 (*n* = 5), CCL20 (*n* = 5), CXCL8 (*n* = 5), CXCL9 (*n* = 5), CXCL10 (*n* = 9) and CXCL11 (*n* = 5). One‐way ANOVA was used for all statistical analysis with Tukey's multiple comparisons test (ns = not significant, *P ≤ 0.05, **P ≤ 0.01, ***P ≤ 0.001, ****P ≤ 0.0001). Error bars show standard deviation of the mean.

Given that PBMCs stimulated with anti‐CD3/CD28 produce high levels of IFNγ and TNFα, we determined if IFNγ and TNFα are the main drivers of the observed changes in cytokine and chemokine secretion during co‐culture. CAFs were treated with recombinant IFNγ and TNFα *in vitro*. Treatment with IFNγ and TNFα mirrored changes observed during co‐culture. Notably, like IL6 [7], IL33 was synergistically increased when both IFNγ and TNFα were present (Fig. 3A). We additionally found a synergistic increase in IL1ra secretion, which could affect iCAF formation via the IL1 receptor [6] (Fig. 3A). Another member of the IL6 family, leukaemia inhibitory factor (LIF), is reported to be amongst the top upregulated genes within iCAF subsets [3, 5] and has been shown to regulate the secretion profile in inflammatory fibroblasts via STAT4 [16]. Interestingly, LIF, as well as GM‐CSF, production by CAFs was significantly increased by TNFα treatment (Fig. 3A). We observe a divergent mechanism of action related to chemokine secretion by CAFs. Here, CCL4 and CXCL8 are mainly driven by TNFα, while CCL2, CCL3, CCL20, CXCL9, CXCL10 and CXCL11 are mediated by a synergistic effect of IFNγ and TNFα (Fig. 3B).

To confirm whether IFNγ and TNFα are the main T‐cell‐derived factors driving chemokine secretion by CAFs, we treated CAFs with TCM containing IFNγ or TNFα neutralising antibodies. In a tested subset of analytes, IFNγ and TNFα were drivers of chemokine secretion (Fig. S3). Interestingly, CXCL10 induction by TCM was entirely abrogated by blocking IFNγ, whereas CXCL8 secretion was dependent on TNFα (Fig. S3).

### IFNγ and TNFα inhibit CXCL12 production by NSCLC CAFs

**Fig. 4:**
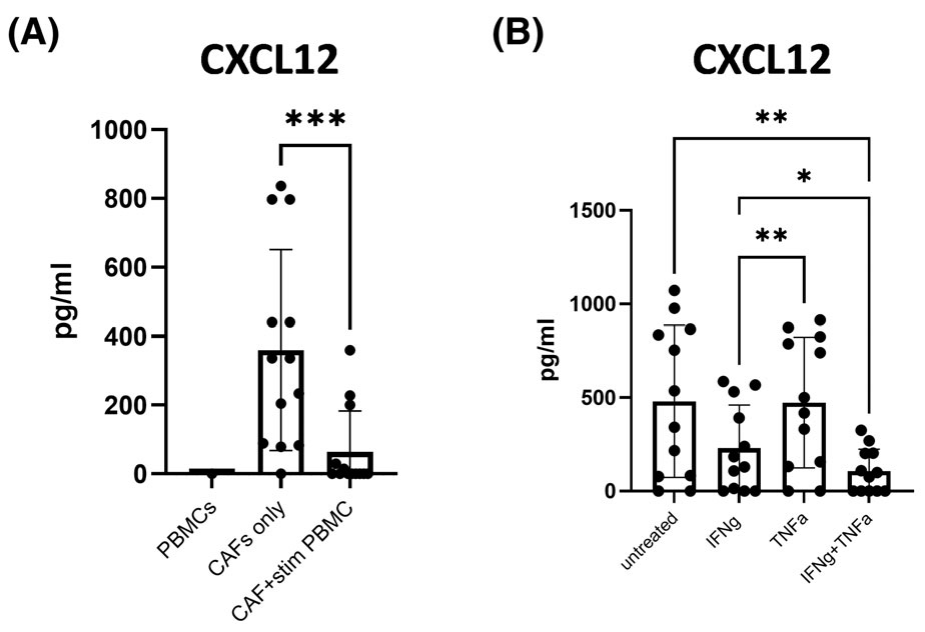
IFNγ inhibits CXCL12 secretion by CAFs. (A) CXCL12 production by PBMCs stimulated with anti‐CD3/anti‐CD28 (PBMCs), NSCLC CAF lines cultured alone (CAFs) or in the presence of anti‐CD3/anti‐CD28 stimulated PBMCs (PBMCs + CAFs). *n* = 10 for PBMC tested in three CAF lines (B) CXCL12 production by CAF lines treated with recombinant rIFNγ (10 ng·mL^−1^), TNFα (25 ng·mL^−1^) or a combination of rIFNγ and TNFα (*n* = 12). One‐way ANOVA was used for all statistical analysis with Tukey's multiple comparisons test (ns, not significant; *P ≤ 0.05, **P ≤ 0.01, ***P ≤ 0.001, ****P ≤ 0.0001). Error bars show standard deviation of the mean.

To further explore the effect of CAF‐T cell crosstalk on the CAF secretome, we assessed levels of CXCL12 production by CAFs in the presence of PBMCs or T‐cell effector cytokines. FAP^+^CAFs are the main source of CXCL12 in the TME [17], and CXCL12 has been repeatedly associated with an iCAF gene signature [18]. CXCL12 has downstream effects on cancer cell proliferation, as well as myeloid cell and T‐cell recruitment and targeting CXCL12 synergises with checkpoint blockade in pancreatic cancer [17]. Our data show that, unlike the other chemokines tested, the secretion of CXCL12 by CAFs is significantly reduced during co‐culture with PBMCs (Fig. 4A). In all 12 NSCLC CAF lines tested, treatment with IFNγ and TNFα reduces CXCL12 secretion *in vitro*, mainly driven by IFNγ (Fig. 4B). These data show that T‐cell effector cytokines IFNγ and TNFα promote the secretion of a number of chemokines by CAFs, while CXCL12 production by CAFs is significantly reduced by IFNγ treatment.

### Cytokine and chemokine‐related genes are upregulated amongst iCAFs in NSCLC tumours

**Fig. 5:**
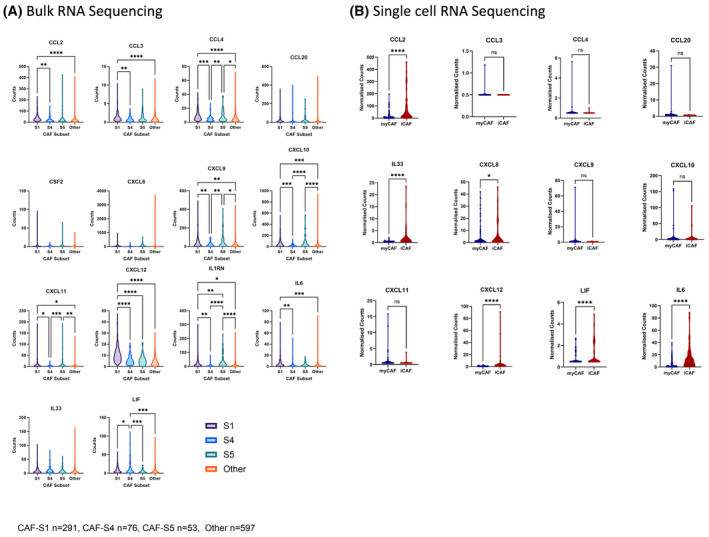
Transcription of cytokine and chemokine genes by CAF subtypes in NSCLC. (A) TCGA data of primary lung cancer (adenocarcinoma (TCGA‐LUAD) and squamous cell carcinoma (TCGA‐LUSC)) comparing cytokine and chemokine transcripts between patients predominant for specific CAF subtypes: CAF‐S1: FAP^high^, PDPN^high^ and αSMA^high^; CAF‐S4: FAP^low^, PDPN^low^ and αSMA^high^; CAF‐S5: FAP^high^, PDPN^high^ and αSMA^low^. CAF‐S1 *n* = 291, CAF‐S4 *n* = 76, CAF‐S5 *n* = 53, Other *n* = 597. (B) ScRNASeq data from Grout *et al*. [12] comparing cytokine and chemokine transcripts between iCAFs and myCAFs. For CSF2 and IL1RN there were no detectable transcripts above baseline between the two groups. One‐way ANOVA was used for statistical analysis with Tukey's multiple comparisons test (ns, not significant; *P ≤ 0.05, **P ≤ 0.01, ***P ≤ 0.001, ****P ≤ 0.0001). Error bars show standard deviation of the mean.

The data presented above shows that effector T‐cell‐derived IFNγ and TNFα can induce and upregulate the secretion of cytokines and chemokines by NSCLC‐derived CAFs cultured *in vitro*. To assess whether these observations hold true within the *in situ* microenvironment of NSCLC, RNA sequencing of NSCLC tumours was assessed for transcription levels of these cytokines and chemokines within CAF subtypes. TCGA bulk RNA sequencing data were divided into patients with predominant CAF subsets previously identified in NSCLC [4]: CAF‐S1: FAP^high^, PDPN^high^ and αSMA^high^; CAF‐S4: FAP^low^, PDPN^low^ and αSMA^high^; CAF‐S5: FAP^high^, PDPN^high^ and αSMA^low^. Bulk RNA sequencing of patient tumours with a predominant CAF‐S1 phenotype show an upregulation of CCL2, CCL3, CCL4, CXCL9, CXCL10, CXCL11, CXCL12, IL1RN, IL6 and LIF, compared to CAF‐S4 which reflects a myCAF phenotype. Similarly, compared to CAF‐S4, CAF‐S5 also upregulates CCL4, CXCL9, CXCL10, CXCL11 and IL1RN suggesting that cytokine and chemokine transcripts are enriched in tumours with high CAF‐S1 and CAF‐S5 and low in the myCAF‐like subset CAF‐S4 (Fig. 5A).

We next assessed RNA transcripts of the cytokine and chemokine panel in a fibroblast single cell RNA sequencing dataset of NSCLC from Grout *et al*. [12] Fibroblasts were defined as myCAFs based on FAP, ACTA2, HOPX, MYH11 and POSTN and iCAFs based on IL6, CXCL12 and PDGFA expression. In addition to IL6 and CXCL12, iCAFs show an upregulation of CCL2, CXCL8, IL33 and LIF illustrating that transcripts of these inflammatory cytokines and chemokines are enriched in iCAF subsets *in situ* (Fig. 5B).

Taken together, these data support that an upregulation of the assessed cytokines and chemokines is found amongst iCAFs in the TME of NSCLC.

## Discussion

Fibroblasts orchestrate immune responses in health and disease, from creating a supportive instructive niche for immune cells, to suppressing immune cell function to prevent an exaggerated immune response. While TGFβ drives a myCAF phenotype, inflammatory CAF differentiation can be mediated via JAK/STAT—and NfKB signalling and a subset of iCAFs in cancer is defined by interferon signalling and termed interferon‐licenced/interferon‐regulated fibroblasts [5, 6, 15, 19]. In human NSCLC, the IFNγ‐iCAF is associated with IFNγ response and cytokine signalling pathways and high expression of chemokines (CCL19, CCL5), as well as MHC II [15]. The magnitude of IFNγ‐producing lymphocyte infiltration has been shown to correlate with the expansion of an ‘interferon‐activated’ inflammatory fibroblast characterised by high expression of CXCL9, CXCL10 and NFkb target genes (e.g. IL6) in other disease contexts [20]. Here, we aimed to investigate whether an ongoing TH1 tumour immune response can similarly promote an inflammatory CAF phenotype in NSCLC. In support of this hypothesis, our data indicate that T‐cell effector cytokines IFNγ and TNFα induce the secretion of cytokines and chemokines by patient‐derived CAFs from NSCLC *in vitro*.

Chemokine signalling regulates the influx and spatial organisation of lymphocytes in the TME and is controlled by cancer intrinsic mechanisms as well as environmental factors [21]. CAFs take part in chemokine signalling indirectly by promoting chemokine secretion by infiltrating immune cells (e.g. CXCL13 by peripheral T helper cells) [7] and directly via secreting a plethora of chemokines in response to environmental cues [2]. For instance, in response to cancer cell conditioned media, CAFs upregulate expression of chemokines such as CCL2 [2], which, together with CCL3 and CCL4 can recruit myeloid cells to the TME. Interestingly, FAP^+^CAF‐secreted CCL2 has previously been shown to activate myeloid‐derived suppressor cells (MDSC), resulting in tumour growth and proliferative suppression of T cells in colorectal cancer [14]. Our data show that while untreated CAFs from NSCLC secrete no CCL2, CCL3 and CCL4, *in vitro* treatment with IFNγ and TNFα induces particularly high levels of CCL2 (~ 100 000 pg·mL^−1^) as well as CCL3 and CCL4, which could result in recruitment of myeloid cells to the TME. Similarly, we also see an induction of high CXCL8 by TNFα, which promotes neutrophil migration into the TME, and is thought to promote tumour progression and CXCL8 expressing CAFs are associated poor prognosis in gastric cancer [14]. We also observed a significant increase in CCL20 secretion by IFNγ and TNFα treated CAFs, which has been implicated in regulatory T‐cell migration to the TME of NSCLC [22] and could therefore also have immunosuppressive consequences. Notably, however, our data also illustrates a strong induction of TH1‐type chemokines CXCL9, CXCL10 and CXCL11, which are ligands for CXCR3 and critical to drive T‐cell recruitment to the tumour sites [6, 21]. Particularly the striking levels of CXCL10 could result in attraction of effector CD8^+^ T cells which is associated with improved survival and positive responses to ICI [1]. Amongst the chemokines tested, CXCL12 secretion followed an opposing pattern, where we observed high secretion in untreated CAFs, which was inhibited by IFNγ treatment. FAP^+^CAF‐derived CXCL12 binds to its receptor CXCR4 commonly expressed by T cells and thereby contains T cells in stromal areas. Inhibition of this chemokine axis leads to a redistribution of T cells and improved immune responses in a number of solid tumours [23]. Additionally, CXCL12 can play a role in recruitment of monocytes, MDSCs and neutrophils and thereby promote the shift towards an immunosuppressive environment [23]. CXCL12 expression is modulated by prostaglandin and TGFβ [24], and we have extended this to show that it is negatively regulated by IFNγ. Lower levels of CXCL12 as a result of IFNγ signalling could therefore significantly re‐shape the TME of NSCLC and influence the localisation of T cells within NSCLC tumours. Blocking CXCL12 signalling has been implicated in the success of immune checkpoint inhibitors in preclinical models and a clinical trial of pancreatic ductal adenocarcinoma, suggesting that intratumoural levels of CXCL12 may have implications within the clinical setting of treatment for patients [25]. Interestingly, multiple studies have shown that high CXCL12 expression is part of an iCAF profile [18, 26], and our analysis of TCGA data shows that CXCL12 is highest amongst NSCLC patients with predominant CAF‐S1. While CAFs treated with IFNγ and TNFα secrete high levels of some chemokines described in iCAF transcriptional profiles, the opposite was observed for CXCL12. In the TME, CAFs are exposed to complex cytokine signalling pathways which is not fully represented in our *in vitro* setup. Subpopulations of CAFs are largely identified by clustering of transcriptional profiles from primary tissue [15], which poses a limitation, as this only shows a snapshot of CAF populations at the time of resection, and it is not fully understood whether these subtypes represent phenotypically distinct populations or a range of activation states that are governed by environmental stimuli and dynamic over time. CXCL12 could be highly expressed amongst iCAFs *in situ* which also upregulate interferon‐related pathways, but our data show that *in vitro*, CXCL12 secretion by CAFs is inhibited by IFNγ in the absence of other environmental factors present in the tissue.

Previous work has shown that CAF‐T cell crosstalk strongly promotes secretion of the IL6 family cytokines IL6 and IL27 by CAFs *in vitro* [7]. IL6 is the main defining feature of inflammatory CAF populations [3, 5, 6, 23, 26]. It is a pleiotropic cytokine with divergent downstream effects in the TME, ranging from fibroblast activation and supporting cancer cell survival to promoting antitumour T‐cell immunity. In lung cancer, CAFs are a major source of IL6 and CAF‐derived IL6 promotes metastasis via STAT3 signalling [2]. Interestingly, we also observe high secretion of the IL6 family member LIF by CAFs treated with TNFα. LIF is overexpressed in cancerous tissue compared to noncancerous lung and associated with poor outcome in lung cancer [27]. Nguyen *et al*. [16] have previously reported that TNF in combination with IL17 induces LIF production by inflammatory fibroblasts in rheumatoid arthritis, and LIF in turn drives an autocrine feedback loop of fibroblast activation via STAT4 resulting in high secretion of IL6 as well as other key inflammatory markers such as IL33, IL8, IL11, IL1α and IL1β. Similarly, LIF activates JAK/STAT signalling and promotes an iCAF phenotype in pancreatic stellate cells (PSC) *in vitro*, and this is induced by IL1 signalling via IL1R which is expressed by iCAFs [5]. Interestingly our data show that CAFs begin to secrete IL1ra when treated with IFNγ and TNFα. This could be a feedback mechanism to inhibit repeated IL1 signalling to prevent an exaggerated inflammatory response. Interestingly, we see that IFNγ and TNFα also induce GM‐CSF secretion by CAFs, which can also be induced via IL1β [26]. Finally, following co‐culture with activated T cells and IFNγ and TNFα, we also observed an increase in IL33 secretion which has also been found to be expressed by iCAFs. This could have negative consequences for the TME, as CAF‐derived IL33 has been associated with driving a type 2 inflammatory response and facilitating metastasis in lung metastasis of breast cancer [28]. In NSCLC, IL33 is locally elevated compared to adjacent noncancerous lung tissue and correlates positively with TNM stage and a murine model has shown that IL33 promotes outgrowth and metastasis of NSCLC. Therefore, an increase in IL33 as a result of CAF‐T cell crosstalk could potentially negatively affect the antitumour response in NSCLC [29].

Our data show that in addition to known iCAF drivers such as IL1 [5] and IFNβ [19], IFNγ and TNFα are able to induce a range of cytokines and chemokines associated with an iCAF phenotype in NSCLC patient‐derived CAFs *in vitro*, which suggests that activated T cells can modulate the CAF secretome. It is unclear, whether IFNγ and TNFα can act as an initial trigger to induce iCAF activation which is then sustained by autocrine amplification loops as previously described for LIF‐LIFR, or whether a continuous exposure to these stimuli is needed to promote this secretory profile.

This study poses two main limitations. Firstly, the CAFs used in this study are grown out *in vitro* from single cell suspensions of NSCLC tumours without prior enrichment in the absence of the many cell signalling pathways and niche pressures present in the TME. As such, the CAFs grown *in vitro* are a uniform population which does not represent the distinct CAF subpopulations that exist in human tissue and it would be of interest to assess the effect of IFNγ and TNFα on different CAF subtypes in NSCLC which should be subject of future work. Secondly, the PBMCs and T cells used in this work are not autologous to the NSCLC patient‐derived CAFs which could impact the CAF response. We have previously shown that we observed no differences in cytokine production of CAFs in response to nonautologous and autologous T cells in this setting [7]; however, it would be beneficial to assess this further in regards to the analytes tested in this study in the future. Furthermore, the *in vitro* model is a reductionist approach to specifically assess the interaction between T‐cell‐derived effector cytokines and the CAF secretome and does not fully capture the complexity of the TME. Considering this interaction does not happen in isolation, it would be desirable to develop more complex *in vitro* models and assess the effect of T‐cell effector cytokines on the CAF secretome within the complexity of the TME.

To conclude, we propose a scenario, in which effector cytokines IFNγ and TNFα derived from lymphocyte infiltrates in hot tumours are able to modulate the CAF secretome and thereby shape the CAF landscape in the TME of NSCLC, which could have implications in immune cell recruitment and activation and ultimately tumour progression and therapy response.

## Author contributions

LK and ARA contributed to the conception and design of the study. LK wrote the first draft of the manuscript and undertook all experimentation and analysis. LK, LM, LN and RAO processed patient samples. ARA undertook supervision, provided funding and reviewed the manuscript. All authors approved the manuscript.

### Peer review

The peer review history for this article is available at https://www.webofscience.com/api/gateway/wos/peer‐review/10.1002/1873‐3468.15083.

## Supporting information


**Fig. S1.** CAF‐T cell interactions increase secretion of cytokines and chemokines (NSCLC patient PBMCs).
**Fig. S2.** CAF‐T cell interactions increase secretion of cytokines and chemokines (Healthy donor purified T cells).
**Fig. S3.** Chemokine secretion induced by supernatant from T cells stimulated with anti‐CD3/anti‐CD28 can be inhibited by neutralising IFNγ and TNFα.

## Data Availability

Data used in this study are available from the corresponding author upon reasonable request.
