# Supplementary material for: IFNγ and TNFα drive an inflammatory secretion profile in cancer‐associated fibroblasts from human non‐small cell lung cancer
Source: FEBS Lett. 2025 Jan 1;599(5):713–23. doi: 10.1002/1873-3468.15083 (PMC11891421; doi:10.1002/1873-3468.15083)
Supplement: Supplementary file 1 — Fig. S1. CAF‐T cell interactions increase secretion of cytokines and chemokines (NSCLC patient PBMCs). Fig. S2. CAF‐T cell interactions increase secretion of cytokines and chemokines (Healthy donor purified T cells). Fig. S3. Chemokine secretion induced by supernatant from T cells stimulated with anti‐CD3/anti‐CD28 can be inhibited by neutralising IFNγ and TNFα. [file FEB2-599-713-s001.docx]

# Supplementary Materials for

**IFNγ and TNFα drive an inflammatory secretion profile in cancer associated fibroblasts from human non-small cell lung cancer**

Lilian Koppensteiner, Layla Mathieson, Liam Neilson, Richard O’Connor, Ahsan R. Akram

**^Supplementary Figures 1-3^**


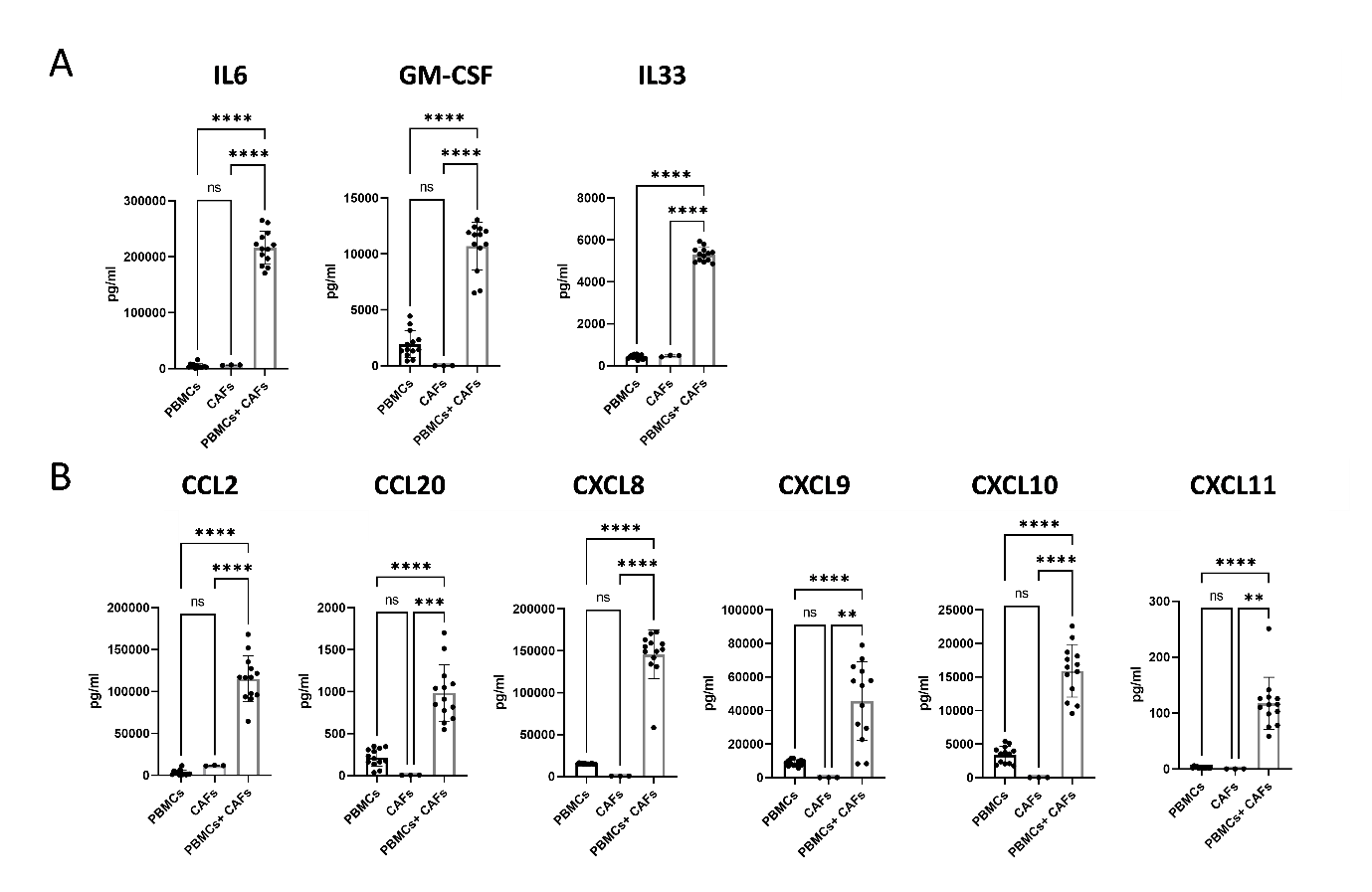


**Supplementary Figure 1 CAF-T cell interactions increase secretion of cytokines and chemokines. (NSCLC patient PBMCs)** PBMCs from early NSCLC patients were stimulated with anti-CD3/ anti-CD28 (PBMCs). One NSCLC CAF line was cultured alone (CAFs) or in the presence of anti-CD3/ anti-CD28 stimulated PBMCs (PBMCs + CAFs). (A) Production of IL6, IL33 and GM-CSF production and (B) CCL2, CCL20, CXCL8, CXCL9, CXCL10 and CXCL11. One-way ANOVA was used for all statistical analysis with Tukey’s multiple comparisons test (ns= not significant, * p≤0.05, ** p≤0.01, *** p≤0.001, **** p≤ 0.0001). n=13 for PBMC tested in 1 CAF line.


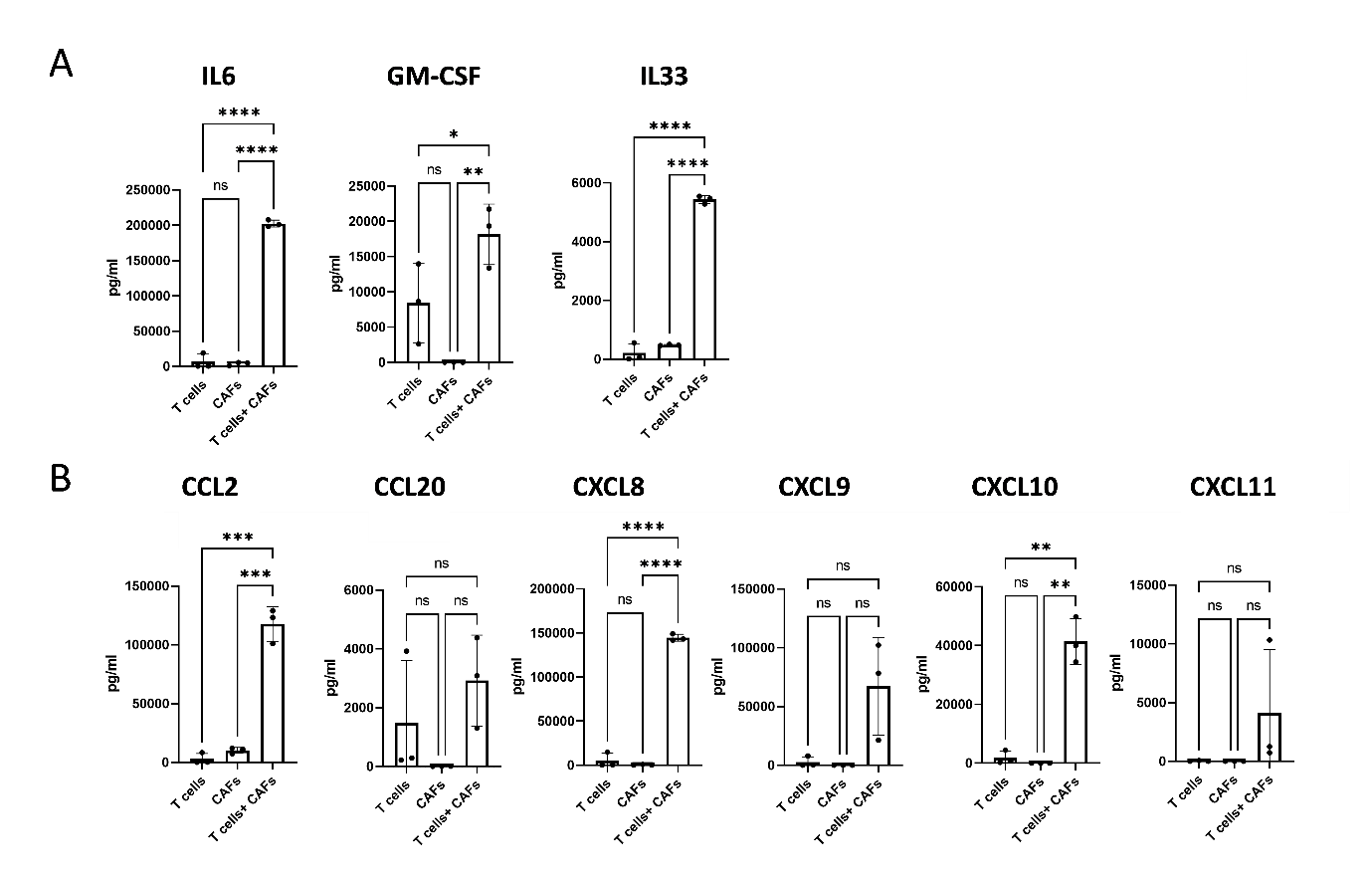


**Supplementary Figure 2 CAF-T cell interactions increase secretion of cytokines and chemokines (Healthy donor purified T cells).** Purified T cells from PBMCs of healthy donors were stimulated with anti-CD3/ anti-CD28 (T cells). Three independently generated NSCLC CAF lines were cultured alone (CAFs) or in the presence of anti-CD3/ anti-CD28 stimulated purified T cells (T cells + CAFs). (A) Production of IL6, IL33 and GM-CSF production and (B) CCL2, CCL20, CXCL8, CXCL9, CXCL10 and CXCL11. One-way ANOVA was used for all statistical analysis with Tukey’s multiple comparisons test (ns= not significant, * p≤0.05, ** p≤0.01, *** p≤0.001, **** p≤ 0.0001). n=3 for PBMC tested in 3 CAF lines.

**
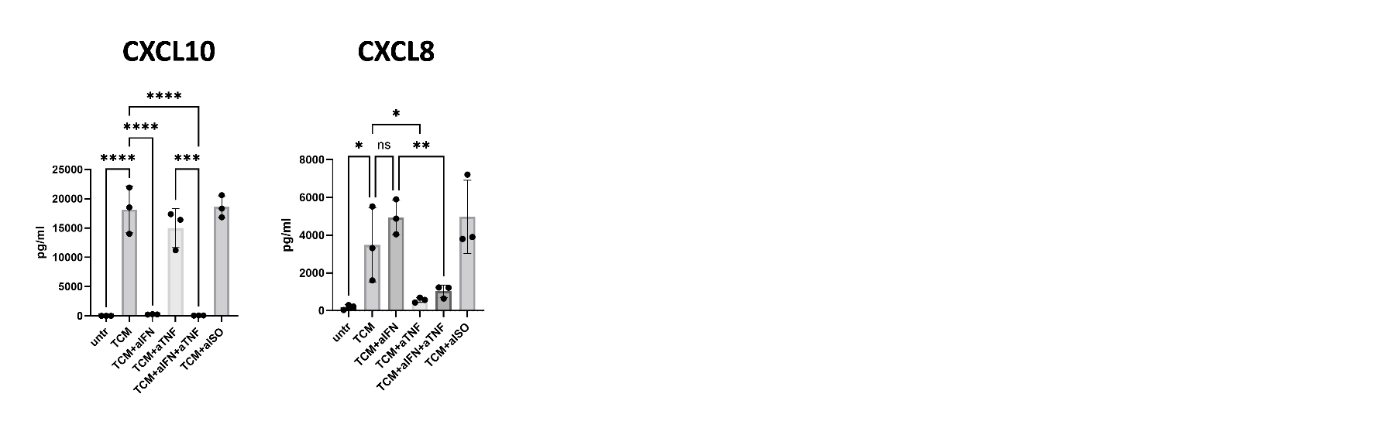
**

**Supplementary Figure *3* Chemokine secretion induced by supernatant from *T cells* stimulated with anti*-C*D3/anti*-*CD28 can be inhibited by neutralising IFNγ and TNFα.** CXCL10 and CXCL8 production by three independently generated NSCLC CAF lines cultured alone, treated with T cell conditioned media with the addition of neutralizing antibodies to IFNγ and/or TNFα or of appropriate isotype matched control antibodies as indicated. One-way ANOVA was used for all statistical analysis with Tukey’s multiple comparisons test (ns= not significant, * p≤0.05, ** p≤0.01, *** p≤0.001, **** p≤ 0.0001) n= 3 CAF lines.
